# Cases

**Published:** 1884-02

**Authors:** E. W. Berridge

**Affiliations:** London


### CLINICAL BUREAU.

### CLINICAL CASES.

E. W. Berridge, M. D., London.

(1) *Berber., Canth., Dig., and Petrol, in Dysuria.*—April 8th, 1874.—Mrs. L. fourteen days ago had to wait six hours without urinating; since then has had the following symptoms: shooting throbbing pain from left inguinal region to pit of stomach; down front of left thigh to knee and to left renal region. Constipated; only two stools in last fourteen days, and then they were scanty, in hard lumps, and difficult, causing pain in anus; once she had to strain for an hour before she could complete the evacuation. Head confused. Pulse, 120. No appetite; thirsty; urine very seldom and scanty, turbid after standing, and high-colored. She has taken *Acon.*, *Bell.*, *Nux*, *Sulph.*, and *Puls.*, all in the third dilution, but without effect. So much for the practice of "Domestic Homœopathy."

In the *Cypher Repertory*, page 459, I found that *Berberis* was the only remedy having pains going from abdomen to back, kidneys, and thighs. At page 462 I found the following symptoms apparently considerably abridged but yet sufficient for the present selection: "Shooting, jerking, or forcing pain, extending *from groin to testicles* and *front of thigh*, and *upward to renal region*;" it had also "shooting pains worse by pressure, extending from right hypochondrium *toward stomach.*" *Berberis*$^{70m}$ (Fincke) every three hours.

11th.—Shooting to stomach and knee gone; still shooting from hypogastrium to back. Shooting downward in hypogastrium. Cutting pain in urethra before and during urination. Tongue brown. Pulse, 96. Thirsty; more appetite; constant rumbling in abdomen. Stool every day, nearly natural, rather soft and light-colored. Heavy pains in kidneys, better when lying on back. Urine has a slight red sediment after standing, but less than before; she urinates a little every half hour.

The cutting in urethra before and during urination was found only under *Canth.* (Bœnninghausen's *Repertory*), and as this was the latest symptom, I took it as the keynote. *Canth.*$^{cm}$ (Fincke) every three hours.

16th.—Cutting in urethra much less; shooting downward in hypogastrium is a little better; it is most marked on left side. Tongue clean; less rumbling. For three days scanty stool directly after liquid food. Pain in kidneys still; head confused; shooting from hypogastrium to back. Has taken no medicine since 13th.

The *Cypher Repertory* gives shooting downward in hypogastrium under *Dig.* and *Verbasc.* The latter was contra-indicated because it has shooting on the *right* side. The *Dig.* symptom I cannot find in Allen. *Dig.*$^{3m}$ (Jenichen) every four hours.

18th.—Has taken only four doses. About ninety minutes after each dose had aggravation of all the pains for two hours. Shooting to knee returned for two hours, but has now ceased. Griping directly after liquid food, followed by diarrhœa; tongue rather brown; sleep not good last night; otherwise unchanged from last report. No medicine for twelve hours. *Dig.*$^{cm}$ (Fincke) one dose.

25th.—Dull pain in loins still, but very much less. Vegetable and liquid food cause griping and diarrhœa. The shooting in left groin is much less, and there is no throbbing. Tongue cleaner. Sleep good. Appetite poor. Urine more copious at night, passing a good amount each time; by day it is scanty and passed seldom; and she has to wait before it passes; this last symptom has existed for three or four days. Head confused.

The *Dig* $^{cm}$ had antidoted the aggravation caused by the *Dig.*$^{3m}$, and improved the condition of the patient, but the painful diarrhœa continued, and there was a new urinary symptom.

*Bry.* and *Petrol.* have diarrhœa from cabbage (Bell's *Repertory*), which was the nearest symptom discoverable; and of these only *Petrol.* has the waiting before urination. I gave *Petrol.*$^{3m}$ (Jenichen) three times a day. All the symptoms ceased in two days, and there was no return July 9th, 1879.

*Cinchona in Erysipelas.*—August 12th, 1881.—Baby seven months old. For one day has had red, elevated spots on body, especially on legs. Left *calf swelled, hard,* and hot, shining pale red; the swelling extending down to back of ankle.

*Cinchona*$^{cm}$ (Fincke) every four hours; first dose at 11 P. M. (Selected from C. Lippe's *Repertory*, pp. 231–2.)

August 13th.—Has had three doses. This morning not nearly so hard, still swelled but less; no redness, much less heat. Spots much less. Had two more doses this day, and by evening was nearly well. Next day was quite well.

August 16th.—Last night hard, hot, red swelling on left forefinger, spreading over back of hand up to elbow. Took another dose last night, and one this morning; it is now better, but since this morning the whole of left external ear is swelled, and also the back of right hand. Gave a dose every four hours.

August 18th.—Improved the same day, much better yesterday; this morning quite well, and remained so.

*Veratrum in Diarrhœa.*—August 9th, 1878.—G. W. A., æt. three, has had diarrhœa for three weeks; three or four stools, sometimes more, every day; stools dark clay-colored, rather slimy, very offensive; before stool, becomes *cold in face,* with *cold sweat on face and forehead.* Diarrhœa is worse from the time of rising till 10 A. M.; then better till 3 P. M., when it gets worse again. It is worse directly after food. It came on with the hot weather, and was better during one or two cool days. He has had allopathic treatment for a week without benefit, the last dose being taken four days ago; then his mother gave him strong tincture of *China,* which made him worse. *Veretrum album*$^{cm}$ (Fincke) one dose.

The child was much better the next day; quite well in a week, and remained well.

July 24th, 1874.—Miss F. has been ill for ten months, with lassitude, coated tongue, malaise, nausea, and diarrhœa. She has been getting worse for a month or more. Now she has vomiting, first of food, then of yellow, bitter bile, but without pain. (This is the latest symptom.) Liquid diarrhœa, which now occurs always with the vomiting, as well as at other times; pain in lower abdomen before diarrhœa, except when she vomits, and then the diarrhœa is painless. *Exhausted after vomiting.* Once, after much straining, there was *froth in the vomit.* *Veratrum album*$^{cm}$ (Fincke) one dose.

August 7th.—Much better. No vomiting after the dose; diarrhœa much better till sixth day, when it returned for three days, but better than before; it still exists, but less than at first, much less liquid, and much less pain before stool. Appetite, sleep, and strength much better. Tongue less coated.

After this there was an *occasional* return of the symptoms, but she then became quite well, and has remained so.

The vomiting, the *latest* symptom, was the *first* to disappear, as HAHNEMANN teaches.

*Lycopodium in Ulcerated Throat.*—January 18th, 1882.—Miss S. had swelling of *right* cervical glands; this disappeared and she then felt swelling in *left* throat, with difficult swallowing. Now there is a whitish ulcer in left tonsil, with soreness there, and swelling of left cervical glands; *hot drinks make throat smart,* but not cold drinks. Feels worse in afternoon, *commencing at* 4 P. M. Has been ill one week. *Lycopod*$^{cm}$ (F. C.) every four hours.

January 24th.—Throat was better on morning of nineteenth, nearly well on twentieth, quite well on twenty-first. As soon as throat began to improve (on nineteenth) she had pains in left leg, worse in the calf, as if torn away ; the pains were felt directly she stood still, but on moving they went away, and it only felt stiff; *the pain was worse at* 4 P. M.; it was removed by a hot bath, remained well.

